# Supplementary material for: Chromothripsis during telomere crisis is independent of NHEJ, and consistent with a replicative origin
Source: Genome Res. 2019 May;29(5):737–49. doi: 10.1101/gr.240705.118 (PMC6499312; doi:10.1101/gr.240705.118)
Supplement: Supplemental Material [file supp_gr.240705.118_Supplemental_file_1.zip › contigs/annotated_contigs/DB78/contig.2.DB78_length_673_mean_cov_15.3640416048.docx]

**DB78_length_673_mean_cov_15.3640416048**

```
CTGTTTAATTTTTAAATATACTGATCATAATTTACTAGACTGATTGTATAAAATCTTAGTGTCATGACTTTAACCTAGTATAGAAAAAA
 >chr14:40816008-40816390 - E=4e-215 p=2e-02
ATAGTAGGATCGTCTTTTCTTTTCAATTTAGTAGATAATTCCCTGTTTTTTCACACTACTCTGGATTATTATGGATGGATGACTCATGA

ATTCTATGCATTCAAAAACTAATATTCTTAGAAGCAGAGAAAAATTTCTTAAAATGAGGCCATTGAAGTACAATGCTTACTTGTCATTT

GGCTTACAAGTATCAGAAATAAGCATCAATGAACTAAGAATGTATTCTCAAGATGAAATACTTTTTCCTAATCTCATAGCTGTCAGACT

ACTATAGAATAGAAACTAATGCTTG|T|GTCTCAGGGATGTCTTTATTCCTATTTTAAAGCCATACATAATTTTTAAAAGTTTTCTTTC
                          >chr14:40808702-40808994 - E=6e-164
CTCCTGTCTCTTAGGCTCCTGTGAGTGAGATAAATATTAAATGAGAAAAAAACATATTTTCAGATGTTTATAATTTCTGAGAACAAAGT

GGACAATATGTCATGAGATAGTAACCTGGATTAACTTGATAGGGAGAAAGGGTGTTCTCCCTGTTATAAATAAGATGATTAAGAATAAC

AAAGCTCTCTTGGTTGGCTACCTAAAGAAGGTAAATGAGTATTCTCAGGCTG
```
